# Signal transducer and activator of transcription family is a prognostic marker associated with immune infiltration in endometrial cancer

**DOI:** 10.1002/jcla.24315

**Published:** 2022-03-03

**Authors:** Xin‐Ying Zhou, Hai‐Yan Dai, Hu Zhang, Jian‐Long Zhu, Hua Hu

**Affiliations:** ^1^ Department of Obstetrics and Gynecology Shanghai Pudong Hospital Fudan University Pudong Medical Center Pudong Shanghai China

**Keywords:** bioinformatic analysis, endometrial cancer, immune infiltration, prognostic markers, STAT family

## Abstract

**Background:**

Signal transducer and activator of transcription (STAT) is a unique protein family that binds to DNA and plays a vital role in regulating major physiological cellular processes. Seven STAT genes have been identified in the human genome. Several studies suggest STAT family members to be involved in cancer development, progression, and metastasis. However, the predictive relationship between STAT family expression and immune cell infiltration in endometrial cancer remains unknown.

**Methods:**

We explored STAT family expression and prognosis in endometrial cancer using various databases. The STRING, GeneMANIA, and DAVID databases, along with GO and KEGG analyses, were used to construct a protein interaction network of related genes. Finally, the TIMER database and ssGSEA immune infiltration algorithm were used to investigate the correlation of STAT family expression with the immune infiltration level in uterine corpus endometrial carcinoma (UCEC).

**Results:**

Our study showed that different STAT family members are differentially expressed in UCEC. STAT1 and STAT2 expression increased at various stages of UCEC, and STAT5A, STAT5B, and STAT6 levels were decreased. STAT3 and STAT4 expression was not significantly different between UCEC and normal tissues. High STAT1 expression may be a prognostic disadvantage of UCEC, and high STAT6 expression may improve UCEC patient prognosis. The STAT family‐associated genes were significantly enriched in signal transduction, protein binding, DNA binding, and ATP binding upon GO analysis. Related genes in the KEGG analysis were mainly enriched in pathways in cancer, viral carcinogenesis, chemokine signaling pathway, JAK/STAT signaling pathway, and regulation of the actin cytoskeleton. In terms of immune infiltration, STAT1 and STAT2 were positively correlated with B, CD8+ T, CD4+ T, and dendritic cells, and neutrophils (*p* < 0.05). All STAT family members were positively correlated with neutrophils and dendritic cells (*p* < 0.05). STAT1 and STAT2 showed similar correlations with all immune cell types, whereas STAT1 and STAT6 showed opposite correlations.

**Conclusion:**

These findings suggest that the STAT family is a prognostic marker, and the immune infiltration level, a therapeutic target, for endometrial cancer.

## Graphical abstract

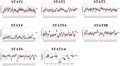

## 1 INTRODUCTION

Endometrial cancer is a common reproductive tract tumor in women, with annual increases in diagnosed cases. With a tendency to develop at a younger age and a high risk of recurrence and death, it gravely endangers women's health, particularly in advanced uterine corpus endometrial carcinoma (UCEC).^1^, ^2^, ^3^ The development of UCEC is a complex process that involves many dysregulated genes.^4^ Despite significant advances in UCEC treatment, including radiotherapy, chemotherapy, and surgical interventions, adjuvant treatment options for patients with endometrial cancer are limited, and 5‐year survival rates remain low owing to the extensive metastasis of advanced UCEC. Therefore, there is a necessity to explore molecular proteins associated with the pathogenesis of endometrial cancer at the gene expression level and identify markers associated with the prognosis and immune infiltration of endometrial cancer, thus providing new therapeutic targets.

Members of the signal transducer and activator of transcription (STAT) protein family are key proteins in cytokine signaling and interferon‐related antiviral activity.^5^, ^6^ These factors have the ability to transmit signals from the cell membrane to the nucleus, thereby activating gene transcription. The main STAT family members identified to date are STAT1, STAT2, STAT3, STAT4, STAT5a, STAT5b, and STAT6.^7^ Their signaling is involved in multiple normal physiological cellular processes, including proliferation, differentiation, apoptosis, angiogenesis, and immune system regulation.^8^ Numerous studies have shown that different STAT family members play essential roles in the development of several cancers, mainly through the JAK/STAT signaling pathway. Flavopereirine inhibits oral cancer progression by inactivating the JAK/STAT signaling pathway via LASP1 upregulation.^9^ IGF2BP3 promotes STAT proteins, which play an important role in cervical cancer development, and JAK/STAT pathway inhibition may be integral in promoting tumor cell death.^11^ Colorectal cancer progression is caused by dysregulation of cytoplasmic transcription factors, including STAT proteins involved in the JAK/STAT signaling pathway.^12^ However, the expression and prognosis of different STAT family members in endometrial cancer and their relationship with the level of immune infiltration in UCEC remain unknown. No studies have reported a bioinformatic analysis of the STAT family in endometrial cancer. Therefore, we comprehensively explored the relationship between the STAT family and endometrial cancer using multiple public databases.

To our knowledge, this is the first study to investigate the relationship between STAT family expression and UCEC immune infiltration in endometrial cancer. Our study helps to identify markers associated with prognosis and immune infiltration in endometrial cancer and is expected to optimize the treatment of patients with endometrial cancer.

## 2 METHODS AND MATERIALS

### 2.1 STAT family expression in pan‐cancer and UCEC

The Cancer Genome Atlas (TCGA) is a landmark cancer genomics project depicting the molecular characterization of over 20,000 primary cancers and providing normal samples of 33 cancer types. Our data were obtained from the TCGA database (https://www.cancer.gov/about‐nci/organization/ccg/research/structural‐genomics/tcga) ALL (pan‐cancer) project and the UCEC project in level 3 HTSeq‐RNAseq data in FPKM format. The data were statistically analyzed and visualized using the R package ggplot2 [version 3.3.3]. We used the UALCAN database (http://ualcan.path.uab.edu/index.html) for analysis of STAT family expression in UCEC.^13^ UALCAN database is an online analysis and mining site based on relevant cancer data from the TCGA database, capable of analyzing the STAT family according to sample type, tumor staging, and the patient race for different subgroup analyses of STAT family expression.

### 2.2 Association between the STAT family and clinical characteristics of UCEC and univariate and multifactor regression analyses

We used RNAseq data in level 3 HTSeq‐FPKM format from the UCEC project in the TCGA database. Statistical analysis and data visualization were performed using the base R package (version 3.6.3). The description of STAT families with UCEC clinical features was performed via Excel tables. We selected only STAT1, STAT2, and STAT6, which are closely related to UCEC clinically. Univariate and multifactor regression analyses for UCEC prognosis used the SURVIVAL package [version 3.2–10] with the prognosis type disease‐specific survival.

### 2.3 STAT family expression in immunohistochemistry

The HPA database (https://www.proteinatlas.org/) provides information on the tissue and cellular distribution of 26,000 human proteins, which uses particular antibodies to examine in detail the distribution and expression of each protein within 64 cell lines, 48 normal human tissues, 20 tumor tissues, and 12 blood cells.^14^ We used the “pathology panel” of the HPA database to detect the expression of different members of the STAT family in UCEC tissues by particular antibodies, and compared it with the expression of different members of the STAT family in normal endometrial tissues in the “tissue panel.”

### 2.4 Survival analysis of the STAT family in UCEC patients

We used the Kaplan–Meier Plotter database (http://kmplot.com/analysis/) to study the association of STAT families with the prognosis of UCEC patients.^15^ The Kaplan–Meier Plotter database is based on gene chips from public databases such as GEO, EGA, and TCGA, and RNAseq data were constructed to assess the impact of 54,675 genes on survival in 21 cancers. When analyzing the predictive value of a specific gene, the Kaplan–Meier Plotter database divides patients into two cohorts based on different quartiles of expression of that gene, and 95% CI and log‐rank *p* values are calculated.

### 2.5 Mutations in the STAT family, the relationship between genes, and protein–protein interaction (PPI) network construction

The cBioPortal database (http://www.cbioportal.org/) is a visual tool for studying and analyzing cancer gene data, which allows analysis of mutations, copy number, and expression of STAT family members in all UCEC samples.^16^ We used UCEC patient data from TCGA to correlate the seven members of the STAT family using Spearman's statistical method. The data were statistically analyzed and visualized using the R package ggplot2 [version 3.3.3].

The String database (https://string‐db.org/) is commonly used to construct protein–protein interaction networks between target proteins, which provides a list of protein molecules that interact with protein regulators based on information from text mining, experimental validation, and raw letter prediction.^17^ We used the String database for PPI–protein interaction network construction for the STAT family.

### 2.6 Gene Ontology (GO) and Kyoto Encyclopedia of Genes and Genomes (KEGG) analyses of STAT family‐related genes

The GEPIA database (http://gepia.cancer‐pku.cn/) is another website that allows dynamic analysis and visualization of TCGA gene expression profile data, which is simple, easy to use, and very powerful.^18^ Using Pearson's correlation analysis, we used the GEPIA database to screen the top 350 genes associated with the STAT family (including the STAT family). The David database (https://david.ncifcrf.gov/) can provide systematic and comprehensive biofunctional annotation information for the large‐scale gene or protein lists, mainly for functional and pathway enrichment analysis of differential genes.^19^, ^20^ We used the David database to perform GO and KEGG analyses on 350 STAT family‐related genes.

### 2.7 Correlation of STAT family gene expression with immune infiltration

We used UCEC patient data in TCGA to statistically analyze and visualize the data using the GSVA package [version 1.34.0] by the ssGSEA immune infiltration algorithm. A lollipop plot of immune infiltration of the STAT family in UCEC was created. The TIMER database (https://cistrome.shinyapps.io/timer) was used to detect immune cell infiltration in tumor tissues using RNAseq expression profiling data.^21^, ^22^ The gene module of the TIMER database demonstrates the gene expression and immune infiltration ratios in relation to each other.

### 2.8 Statistical analysis

All statistical analyses were performed on the respective database sites, and the data were calculated using R software (v.3.6.3). The chi‐squared, Fisher's exact, and Wilcoxon rank‐sum tests were used to analyze clinical information. Statistical significance was set at *p* < 0.05.

## 3 RESULTS

### 3.1 STAT family expression in pan‐cancer and UCEC

**FIGURE 1:**
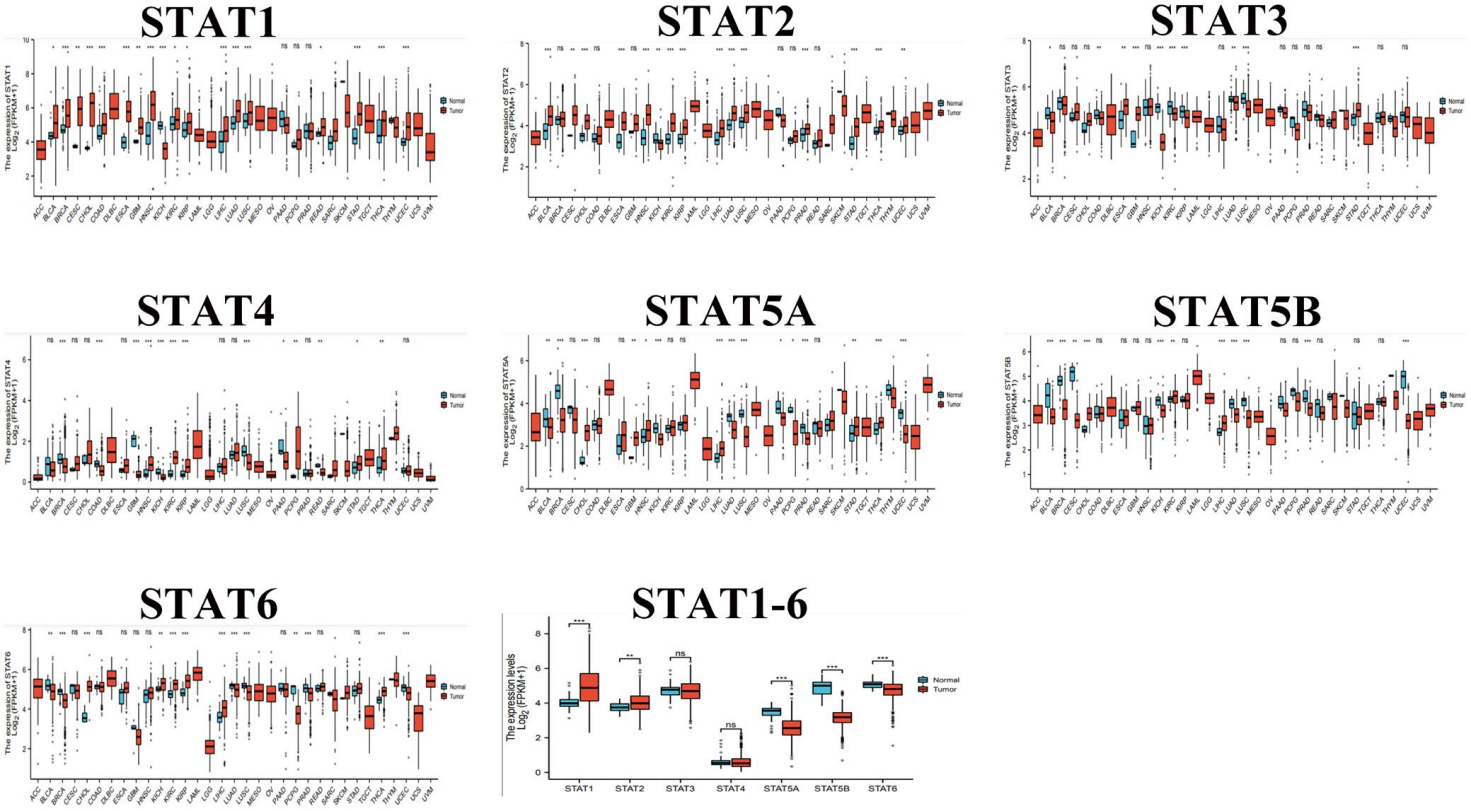
STAT family expression in pan‐cancers (ns, *p* ≥ 0.05; *p < 0.05; **p < 0.01; and ***p < 0.001)

**FIGURE 2:**
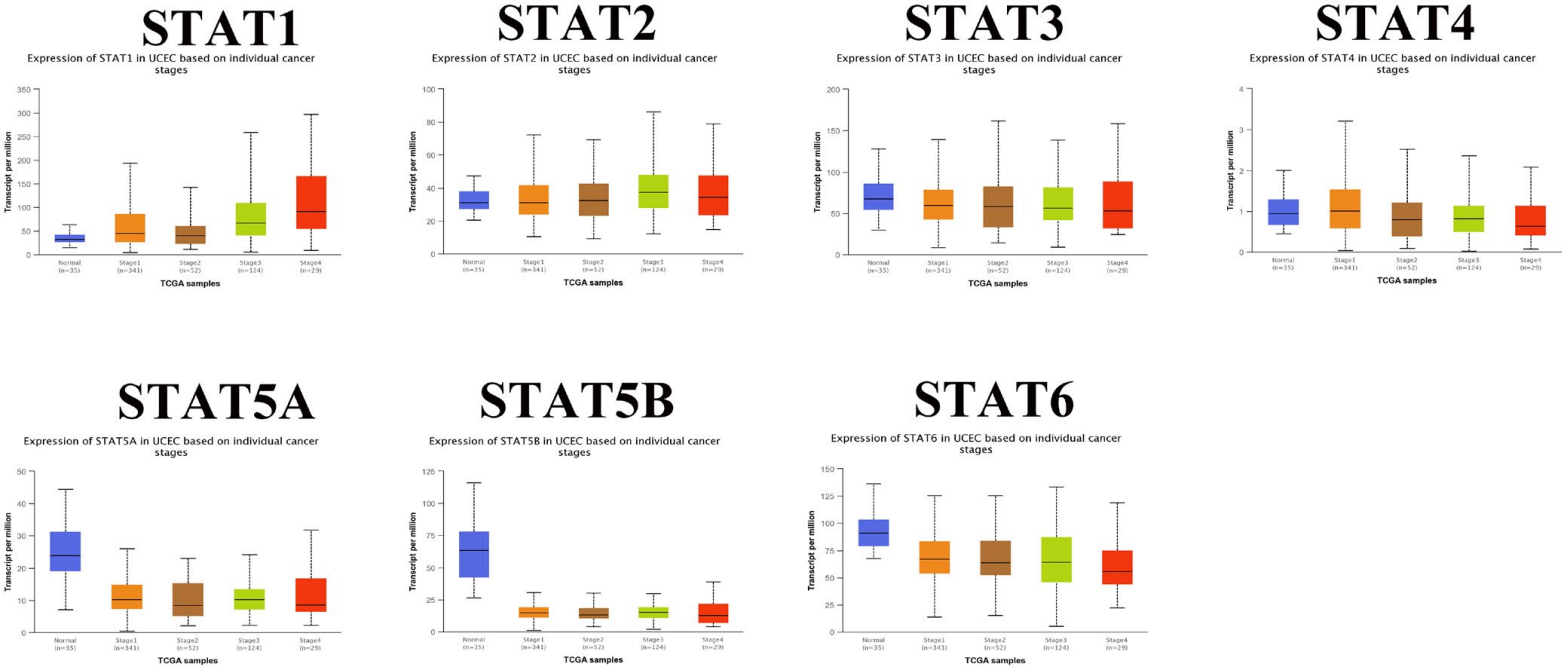
Expression of STAT family in different stages of endometrial cancer (*p*<0.05 for different stages of STAT1 compared with normal tissue; *p* < 0.05 for STAT2 in stage 1 and stage 3 of UCEC compared with normal tissue; and no statistically significant difference in expression for different stages of STAT3 and STAT4 compared with normal tissue. STAT5A, STAT5B, and STAT6 were expressed differently at different stages compared with normal tissues, *p* < 0.05)

We analyzed STAT family expression in pan‐cancer using pan‐cancer tumor data from The Cancer Genome Atlas (TCGA) database (https://www.cancer.gov/about‐nci/organization/ccg/research/structural‐genomics/tcga) (Figure 1). The differences in the expression of different STAT family members in UCEC and normal tissues are shown in Figure 1. STAT1 and STAT2 expression was significantly higher in UCEC. STAT5A, STAT5B, and STAT6 expression was significantly lower in UCEC, while the difference in STAT3 and STAT4 expression between UCEC and normal tissues was not statistically significant. To further analyze the expression of STAT family members in UCEC, we analyzed the differences in STAT family expression at different stages of UCEC using the UALCAN database (http://ualcan.path.uab.edu/index.html; Figure 2). STAT1 and STAT2 were highly expressed at all stages of UCEC (particularly stages 1 and 3 for STAT2). STAT5A, STAT5B, and STAT6 were lowly expressed in all stages of UCEC. In contrast, STAT3 and STAT4 expression in all stages of UCEC was not significantly different from that in normal tissues.

### 3.2 Association between the STAT family and the clinical characteristics of UCEC and univariate and multifactor regression analyses

UCEC data from TCGA were used to describe the relationship between STAT family members and the clinical characteristics of UCEC using Excel. STAT1, STAT2, and STAT6 expression was closely related to the clinical characteristics of UCEC (Table 1). High STAT1 expression was significantly associated with clinical stage, age, histological type, residual tumor, histological grade, and median survival age of UCEC. Similar to STAT1, high STAT2 expression was closely associated with clinical stage, age, histological type, and histological grade of UCEC. Low STAT6 expression was associated with histological type, residual tumor of UCEC, histological grade, and median survival age of UCEC. Next, univariate and multifactorial Cox regression analyses were performed for all STAT family members using the UCEC data in TCGA database (Table 2), with disease‐specific survival selected as the prognostic type. Notably, high STAT1 expression was a risk factor for UCEC prognosis (*p* < 0.05). High STAT6 expression may reduce the risk of UCEC prognosis (*p* < 0.05).

**TABLE 1:** Relationship between STAT family gene expression and clinical features of UCEC

Characteristic	Low expression of STAT1	High expression of STAT1	*p* value	Low expression of STAT2	High expression of STAT2	*p* value	Low expression of STAT6	High expression of STAT6	*p* value
Clinical stage, *n* (%)			< 0.001			< 0.001			0.082
Stage I	191 (34.6%)	151 (27.4%)		188 (34.1%)	154 (27.9%)		160 (29%)	182 (33%)	
Stage II	32 (5.8%)	19 (3.4%)		30 (5.4%)	21 (3.8%)		25 (4.5%)	26 (4.7%)	
Stage III	46 (8.3%)	84 (15.2%)		45 (8.2%)	85 (15.4%)		71 (12.9%)	59 (10.7%)	
Stage IV	7 (1.3%)	22 (4%)		13 (2.4%)	16 (2.9%)		20 (3.6%)	9 (1.6%)	
Age, *n* (%)			0.002			0.021			0.126
<=60	121 (22%)	85 (15.5%)		116 (21.1%)	90 (16.4%)		94 (17.1%)	112 (20.4%)	
>60	153 (27.9%)	190 (34.6%)		157 (28.6%)	186 (33.9%)		181 (33%)	162 (29.5%)	
Histological type, *n* (%)			< 0.001			< 0.001			< 0.001
Endometrioid	242 (43.8%)	168 (30.4%)		238 (43.1%)	172 (31.2%)		192 (34.8%)	218 (39.5%)	
Mixed	11 (2%)	13 (2.4%)		5 (0.9%)	19 (3.4%)		7 (1.3%)	17 (3.1%)	
Serous	23 (4.2%)	95 (17.2%)		33 (6%)	85 (15.4%)		77 (13.9%)	41 (7.4%)	
Residual tumor, *n* (%)			0.025			0.297			0.005
R0	200 (48.4%)	175 (42.4%)		192 (46.5%)	183 (44.3%)		176 (42.6%)	199 (48.2%)	
R1	11 (2.7%)	11 (2.7%)		15 (3.6%)	7 (1.7%)		15 (3.6%)	7 (1.7%)	
R2	3 (0.7%)	13 (3.1%)		8 (1.9%)	8 (1.9%)		13 (3.1%)	3 (0.7%)	
Histologic grade, *n* (%)			< 0.001			< 0.001			< 0.001
G1	67 (12.4%)	31 (5.7%)		58 (10.7%)	40 (7.4%)		35 (6.5%)	63 (11.6%)	
G2	83 (15.3%)	37 (6.8%)		78 (14.4%)	42 (7.8%)		43 (7.9%)	77 (14.2%)	
G3	122 (22.6%)	201 (37.2%)		137 (25.3%)	186 (34.4%)		190 (35.1%)	133 (24.6%)	
Age, meidan (IQR)	62 (56, 71)	65 (59, 71.5)	0.017	63 (56, 72)	65 (59, 71)	0.080	66 (58, 73)	63 (56.25, 70)	0.020

**TABLE 2:** Univariate and multifactor regression analyses of STAT family in UCEC

Characteristics	Total (*N*)	Univariate analysis		Multivariate analysis	
		Hazard ratio (95% CI)	*p* value	Hazard ratio (95% CI)	*p* value
STAT1	549				
Low	275	Reference			
High	274	1.655 (1.001–2.736)	0.049	1.780 (1.074–2.949)	0.025
STAT2	549				
Low	276	Reference			
High	273	1.426 (0.867–2.345)	0.163		
STAT3	549				
Low	274	Reference			
High	275	0.794 (0.481–1.312)	0.369		
STAT4	549				
Low	276	Reference			
High	273	0.751 (0.456–1.238)	0.262		
STAT5A	549				
Low	274	Reference			
High	275	1.176 (0.717–1.928)	0.521		
STAT5B	549				
Low	274	Reference			
High	275	0.981 (0.598–1.609)	0.940		
STAT6	549				
Low	274	Reference			
High	275	0.469 (0.274–0.804)	0.006	0.445 (0.259–0.763)	0.003

### 3.3 STAT family expression in immunohistochemistry

**FIGURE 3:**
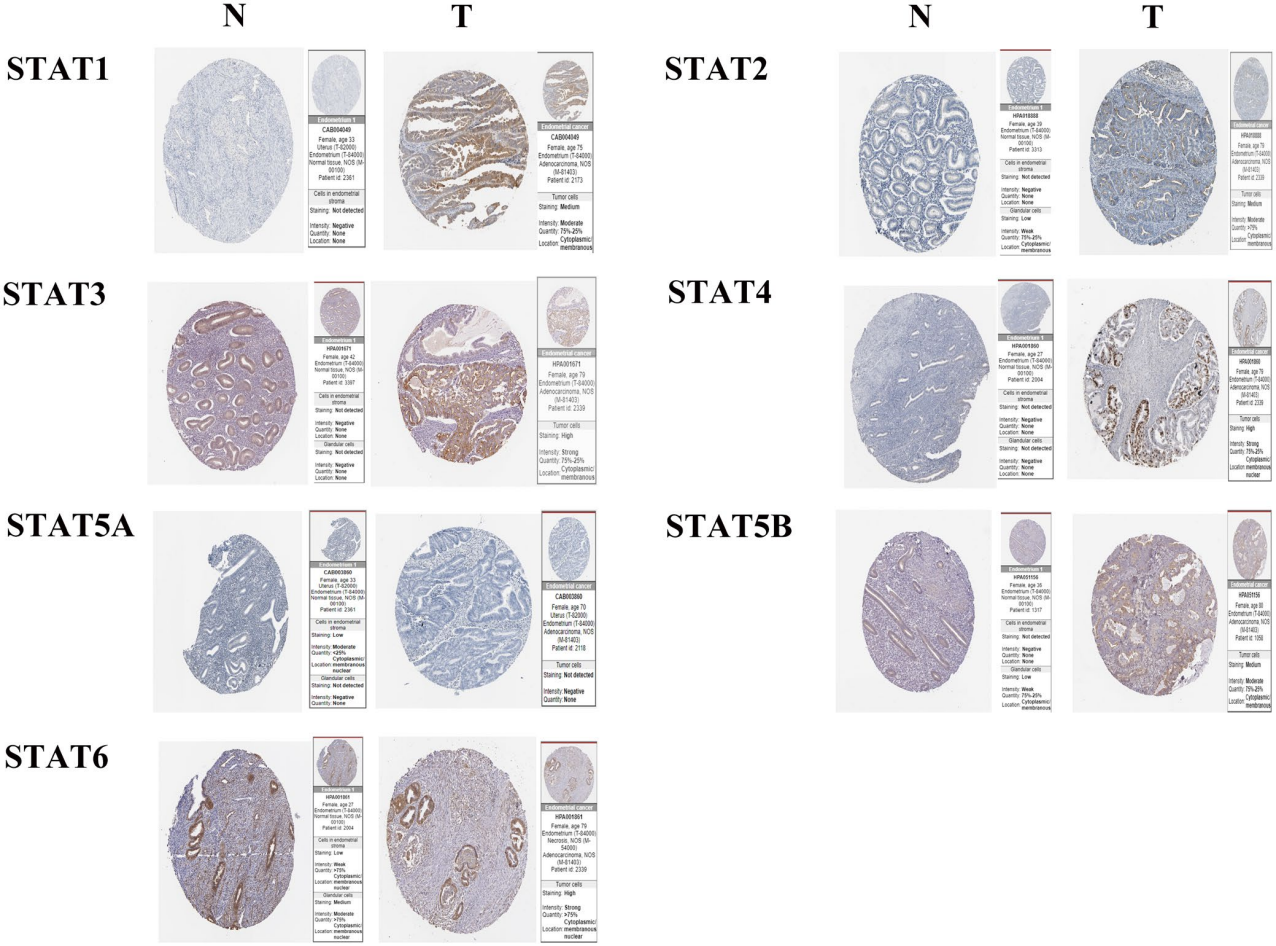
Immunohistochemical expression of STAT family in UCEC

We used the Human Protein Atlas (HPA) database (https://www.proteinatlas.org/) to investigate the differences in STAT family expression between UCEC and normal endometrial tissues (Figure 3). STAT1, STAT2, STAT3, and STAT4 were more significantly expressed in UCEC tissues. STAT5A, STAT5B, and STAT6A were differentially expressed in the normal and UCEC tissues. These results are slightly different from those obtained using the UALCAN database.

### 3.4 STAT family is strongly associated with the prognosis of patients with UCEC

**FIGURE 4:**
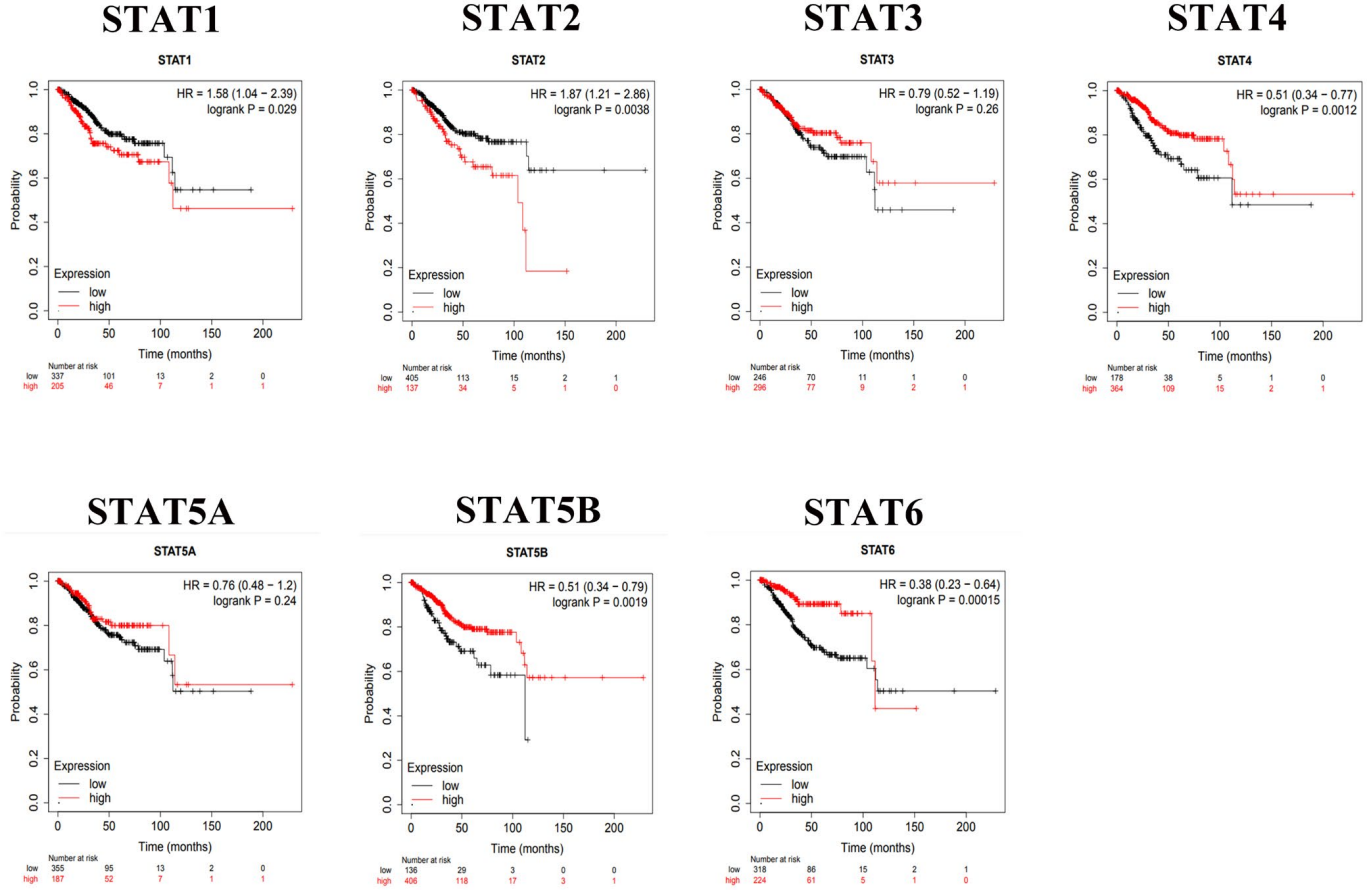
Relationship between STAT family gene expression and OS curve of UCEC.

We used the Kaplan–Meier plotter database (http://kmplot.com/analysis/) to investigate the relationship between STAT family members and the prognosis of patients with UCEC (Figure 4). High STAT1 and STAT2 expression was significantly associated with overall survival (OS) in UCEC (*p* < 0.05), suggesting that STAT1 and STAT2 may be unfavorable factors in UCEC prognosis. High STAT4, STAT5B, and STAT6 expression may significantly improve the prognosis of patients with UCEC (*p* < 0.05). Moreover, STAT3 and STAT5A expression was not associated with the OS curve of UCEC.

### 3.5 Mutations in the STAT family, the relationship between genes, and PPI network construction

**FIGURE 5:**
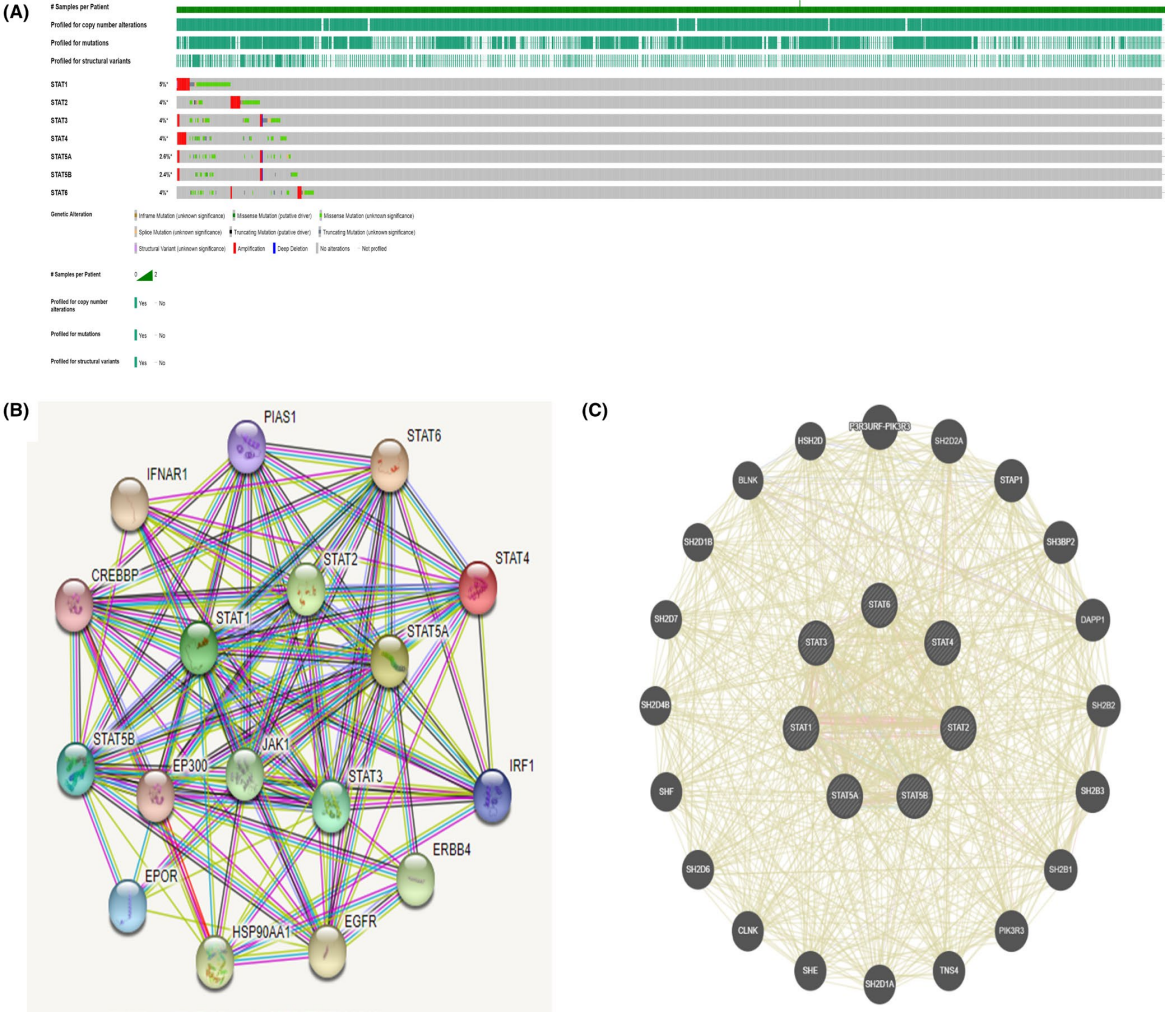
Mutation profile of STAT family and PPI network construction

**FIGURE 6:**
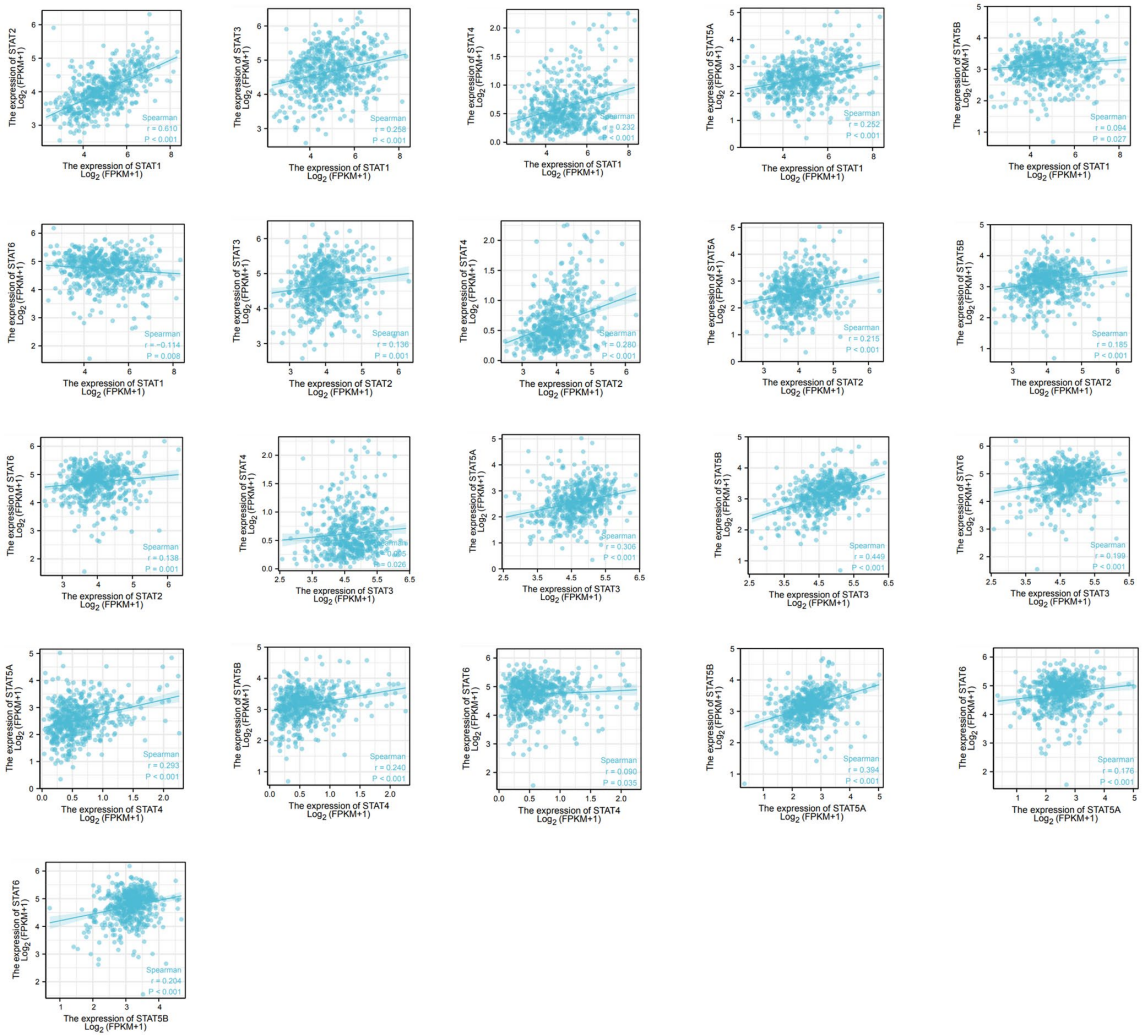
Correlation analysis of STAT family members

The cBioPortal database (http://www.cbioportal.org/) was used to study STAT family mutations in patients with UCEC (Figure 5). The STAT family members were mutated to varying degrees in UCEC (mutation rate <5%). Of 1444 UCEC patient samples, STAT1, STAT2, STAT3, STAT4, STAT5A, STAT5B, and STAT6 were mutated at a rate of 5% (79/1444), 4% (59/1444), 4% (60/1444), 4% (53/1444), 2.6% (38/1444), 2.4% (34/1444), and 4% (52/1444), respectively. We then used data on patients with UCEC from TCGA to correlate the seven members of the STAT family using Spearman's statistics (Figure 6). A positive correlation between the STAT family members (*p* < 0.05) was identified, except for STAT1 expression, which was negatively correlated with STAT6 expression.

The STRING (https://string‐db.org/) and GeneMANIA databases (http://genemania.org/) were used for PPI network construction of the STAT family (Figure 5). The ten genes most closely related to STAT family molecules in the STRING database were selected, namely EPOR, IRF1, PIAS1, CREBBP, EP300, IFNAR1, EGFR, HSP90AA1, ERBB4, and JAK1. Alternatively, the GeneMANIA database demonstrated 20 target genes interacting with the STAT family, with more SH2 signaling protein family genes, suggesting that the STAT family is primarily involved in signal transduction biological processes.

### 3.6 GO and KEGG analyses of STAT family‐related genes

**FIGURE 7:**
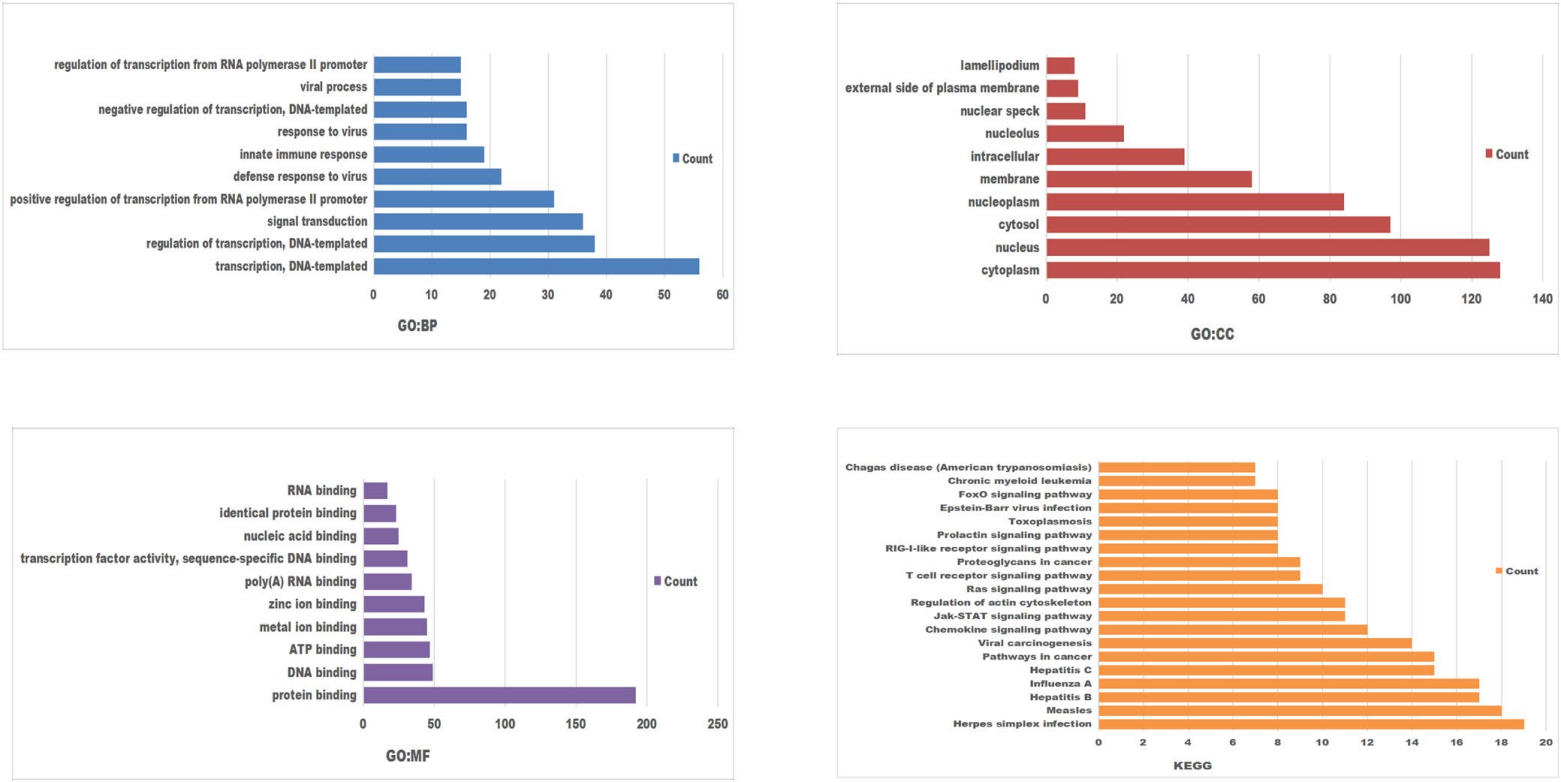
GO and KEGG analyses of STAT family‐related genes

First, the top 350 genes associated with the STAT family (including the STAT family) were screened using the GEPIA database (http://gepia.cancer‐pku.cn/) and Pearson's correlation analysis. These 350 related genes were subjected to GO and KEGG analyses using the David database (https://david.ncifcrf.gov/; Figure 7). The first three items of biological processes in the GO analysis were transcription, DNA‐templated (GO: 0006351); regulation of transcription, DNA‐templated (GO:0006355); and signal transduction (GO:0007165). The top three items in the cellular component analysis were cytoplasm (GO:0005737), nucleus (GO:0005634), and cytosol (GO:0005829). The top three items in the molecular function analysis were protein binding (GO:0005515), DNA binding (GO:0003677), and ATP binding (GO:0005524). In the above GO analysis, *p* < 0.05. The KEGG pathways of STAT family‐related genes were mainly concentrated in herpes simplex infection (hsa05168), measles (hsa05162), hepatitis B (hsa05161), influenza A (hsa05164), hepatitis C (hsa05160), pathways in cancer (hsa05200), viral carcinogenesis (hsa05203), chemokine signaling pathway (hsa04062), Jak‐STAT signaling pathway (hsa04630), and regulation of the actin cytoskeleton (hsa04810).

### 3.7 Correlation of STAT family gene expression with immune infiltration

**FIGURE 8:**
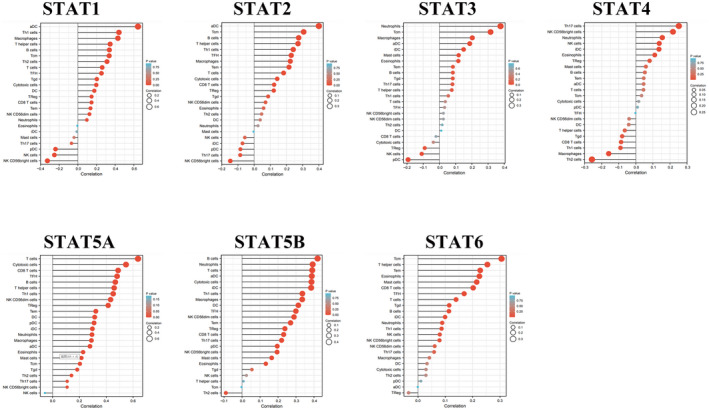
Correlation of STAT family gene expression with immune cell infiltration levels (1) (lollipop plot)

The UCEC patient data from TCGA were used to analyze the correlation between STAT family genes and various immune cell types using the ssGSEA immune infiltration algorithm (Figure 8). STAT1 was significantly positively correlated with aDC, Th1 cells, and macrophages and negatively correlated with natural killer (NK) CD56bright cells, NK cells, and pDC. STAT2 was positively correlated with aDC, Tcm, and B cells and negatively correlated with NK CD56bright cells, Th17 cells, and pDC. STAT3 was positively correlated with neutrophils, Tcm, and macrophages and negatively correlated with pDC, NK cells, and TReg. STAT4 was positively correlated with T cells, cytotoxic cells, and CD8+ T cells and had no significant negative correlation. STAT5A was positively correlated with B cells, neutrophils, and T cells and negatively correlated with Th2 cells. STAT5B was positively correlated with Tcm, T helper cells, and Tem, and there was no significant negative correlation with immune cells. STAT6 was positively correlated with Th17 cells, CD56bright cells, and neutrophils and negatively correlated with Th2 cells, macrophages, and Th1 cells. STAT1 and STAT2 correlated similarly with all immune cell types, whereas STAT1 and STAT6 correlated oppositely with all immune cell types.

**FIGURE 9:**
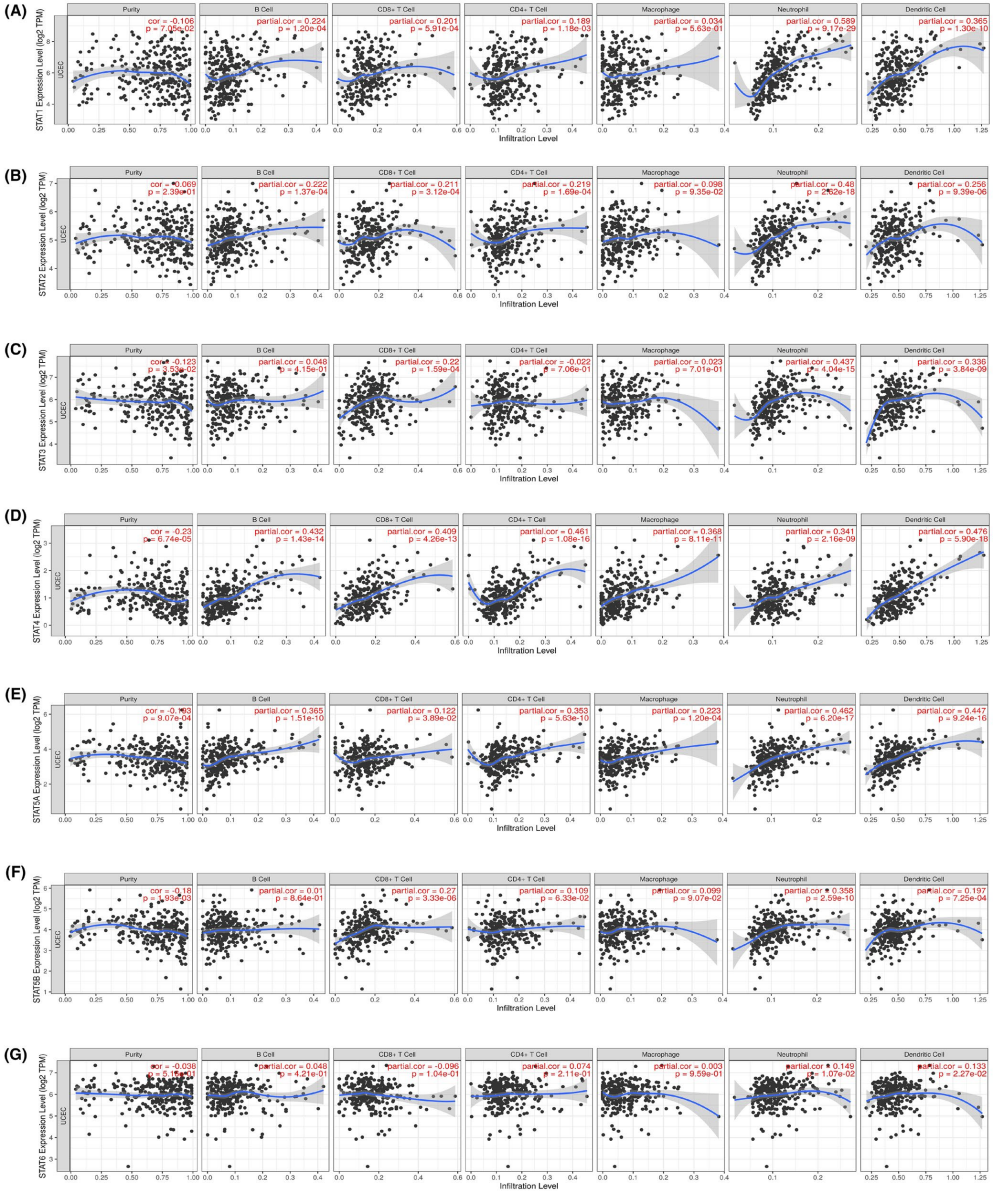
Correlation between STAT family gene expression and immune cell infiltration levels (2) (scatter plot)

The relationship between STAT family gene expression and infiltrating lymphocytes was further analyzed using the TIMER database (https://cistrome.shinyapps.io/timer; Figure 9). STAT1 and STAT2 were positively correlated with B cells, CD8+ T cells, CD4+ T cells, neutrophils, and dendritic cells (*p* < 0.05). STAT3 and STAT5B were positively correlated with CD8+ T cells, neutrophils, and dendritic cells (*p* < 0.05). STAT4 and STAT5A were positively correlated with B cells, CD8+ T cells, CD4+ T cells, macrophages, neutrophils, and dendritic cells (*p* < 0.05). STAT6 expression was positively correlated with neutrophils and dendritic cells (*p* < 0.05). All STAT family members were positively correlated with neutrophils and dendritic cells (*p* < 0.05).

## 4 DISCUSSION

Numerous studies have shown that different STAT family members play essential roles in the development of several cancers, including oral,^9^ bladder,^10^ cervical,^11^ and colorectal cancers.^12^ It has been proposed that the JAK/STAT signaling pathway is involved in the development of UCEC, and targeted inhibition of the IL‐6 receptor and its downstream effectors JAK1 and STAT3 significantly reduces UCEC tumor cell growth.^23^ However, no bioinformatic analysis has been reported on the various STAT family members in endometrial cancer, and whether STAT family expression is associated with tumor immune infiltration in UCEC remains unknown.

This study explored, for the first time, the expression and prognostic value of various STAT family members in endometrial cancer using multiple databases. Pan‐cancer tumor data from TCGA database suggested that STAT family expression was elevated in various tumors, including breast invasive carcinoma, colon adenocarcinoma, liver hepatocellular carcinoma, stomach adenocarcinoma, and UCEC. Among these, different STAT family members are differentially expressed in UCEC. STAT1 tends to be highly expressed in UCEC, whereas STAT6 tends to be lowly expressed in UCEC.

The UCEC data in TCGA showed that the STAT family was strongly associated with the clinical stage, age, histological type, residual tumor, histological grade, and median survival age of UCEC, with statistically significant differences. Univariate and multifactorial Cox regression analyses were performed for all STAT family members. The results showed that only STAT1 and STAT6 were statistically significant (*p* < 0.05). This suggests that STAT1 and STAT6 are more closely related than other members of the STAT family to UCEC prognosis. However, STAT3 and STAT5 have been studied more thoroughly, while the role of STAT1, STAT2, STAT4, and STAT6 in tumor development has been studied less thoroughly.^8^ For example, Wallbillich et al. found that STAT3 expression in endometrial cancer promotes tumor growth and that metformin inhibits STAT3, thereby promoting apoptosis and inhibiting endometrial cancer cell proliferation, which emphasizes the possibility that STAT3 promotes endometrial cancer development.^24^ Immunohistochemical analysis using the HPA database further validated the expression of different STAT family members in UCEC tissues. The expression of STAT1, STAT2, STAT3, and STAT4 was more intense in UCEC tissues. Staining for STAT5A, STAT5B, and STAT6A was higher in normal endometrial tissues, indicating that normal tissues also express STAT5A, STAT5B, and STAT6A. This suggests that STAT5 and STAT6 may not be the factors that promote the development of endometrial cancer.

Results from the Kaplan–Meier plotter database suggest a relationship between the expression of different STAT family members and UCEC prognosis. STAT1 and STAT2 may be detrimental to UCEC prognosis. The hazard ratios (HRs) for STAT1 and STAT2 were 1.58 and 1.87, respectively. The *p*‐values of the OS curves were log‐rank *p* = 0.029 (STAT1) and log‐rank *p* = 0.0038 (STAT2). It has been suggested that STAT1 can directly bind to and activate transcription of the long non‐coding RNA LINC01123 promoter region. High LINC01123 expression is associated with advanced clinical progression and poor clinical outcomes in patients with endometrial cancer, demonstrating that STAT1 promotes proliferation and metastasis in endometrial cancer.^4^ However, Albacker et al. reported that, in solid tumors, activation of STAT3 or STAT5 by mutations or cytokine signaling is protumorigenic. In contrast, STAT1 activation upregulates antigen presentation and has an antitumorigenic effect.^25^ Moreover, our study concluded that high STAT4, STAT5B, and STAT6 expression may be beneficial for improving the prognosis of patients with UCEC. The HR for STAT4 and STAT5B was 0.51, and that for STAT6 was 0.38. The *p*‐values of the OS curves were log‐rank *p* = 0.0012 (STAT4), log‐rank *p* = 0.0019 (STAT5B), and log‐rank *p* = 0.00015 (STAT6). STAT5 may act as a prognostic factor for tumors. Sultan et al. demonstrated that STAT5A induces E‐cadherin and promotes β‐catenin binding to the cell surface through E‐cadherin‐mediated linkage, thereby inhibiting human breast cancer cells.^8^ In another study, increased expression of IL‐4/STAT6 signaling was associated with reduced tumor volume and weight, as well as the increased expression of apoptotic proteins.^26^ However, the role of STAT5 in promoting tumor development has also been documented. For example, JAK2/STAT5B promotes tumor proliferation and metastasis in breast and prostate cancers.^27^, ^28^ STAT6 is also highly expressed in various tumors. STAT6 plays a role in tumorigenesis, immunosuppression, proliferation, and metastasis of human cancers. The highly activated IL‐4/IL‐4Ra/STAT6 signaling in prostate cancer stem cell‐like cells validates the tumorigenic activity of STAT6.^26^ However, the results of these studies contradict our findings. This may be because the role of STAT in cancer is highly dependent on the tumor environment, and its effects are caused by subtle and complex transcriptional modifications between different STAT molecules rather than by a single family member. STAT family members can act as both oncogenes and tumor suppressors. Therefore, the use of the STAT family as a diagnostic and prognostic basis requires careful consideration of each specific cancer type and the characteristics of each patient.^8^, ^29^

To further investigate the mechanism of STAT family development in UCEC, we screened the STRING and GeneMANIA databases for STAT family‐interacting target gene molecules. The ten gene molecules most closely associated with the STAT family in the STRING database were EPOR, IRF1, PIAS1, CREBBP, EP300, IFNAR1, EGFR, HSP90AA1, ERBB4, and JAK1. This suggests that these molecules could be potential upstream and downstream molecules in the mechanism of action of the STAT family members. The synergistic binding of STAT1‐IRF1 and STAT1‐IRF1‐IRF8 plays a key role in inflammation and host defense functions.^30^ Bhattacharya et al. showed that the downregulation of IFNAR1 directly attenuated the antiproliferative, antimigratory, and proapoptotic effects of IFNAR1 in tumor cells. IFNAR1 binds to and activates Tyk2 in the cytoplasmic structural domain while phosphorylating STAT1 and STAT2. In addition to the classical JAK‐STAT pathway, IFNAR1 negatively regulates the STAT3 pathway upon binding to IFN1 to accelerate metastasis in endometrial cancer.^31^ CBP and its highly homologous paralog EP300 (collectively CBP/EP300) belong to the histone acetyltransferase family, which are central players in chromatin remodeling and gene activation in cancer. By regulating H3K27 acetylation of STAT‐related genes, the CBP/EP300 bromodomain controls the function of myeloid‐derived suppressor cells within tumors. Inhibition of bromodomain reduces tumor growth, suggesting that the CBP/EP300 bromodomain may be targeted to enhance antitumor immunity.^32^ herefore, further analysis of the relationship between these related genes and the STAT family could help explore the mechanism of their development in UCEC. Genes of the SH2 signaling protein family are predominant among the target genes interacting with the STAT family, as demonstrated by the GeneMANIA database. This may be related to the structure and function of the STAT family members. STAT proteins consist of several structurally and functionally conserved regions. The Src homology 2 (SH2) domain, together with the N‐terminal domain, mediates homodimerization and heterodimerization of STAT monomers during activation. The SH2 structural domain is highly conserved and is the target of the majority of STAT inhibitors.^8^ Blocking the SH2 domain of the STAT family and inhibiting its phosphorylation and downstream signaling may be useful for the treatment of diseases.^8^ The JAK/STAT signal transduction pathway is the main route of intracellular transmission of most cytokines, and it is through this pathway that the STAT protein family functions and is involved in the development of various tumors.

The cBioPortal database was used to understand the mutation status of the STAT family, and all STAT family members were mutated to varying degrees in UCEC (mutation rate <5%). The highest degree of mutation was in STAT1 (mutation rate 5%), and the lowest degree of mutation was in STAT5B (mutation rate 2.4%). Spearman's statistics were used to correlate the seven STAT family members. Positive correlations were found among the STAT family members, except for STAT1 expression, which was negatively correlated with STAT6 expression. The negative correlation between STAT1 and STAT6 expression in UCEC further confirms that STAT1 and STAT6 play opposing roles in UCEC prognosis. GO and KEGG analyses of STAT family‐related genes were performed using the DAVID database. GO analysis revealed significant enrichment of genes related to transcription, DNA‐templated; signal transduction; protein binding; and DNA binding. STAT is a classical transcription factor that binds directly to DNA regulatory elements and controls the transcription of related genes.^33^ KEGG pathway analysis focused on pathways in cancer, chemokine signaling pathway, and JAK/STAT signaling pathway. STAT functions mainly in a phosphorylated form to bind to DNA to recruit transcription factors and also to interact with cytoskeletal regulators.^27^ This suggests that STAT‐related genes play critical roles in signal transduction and transcriptional activation.

Tumor‐associated immune‐infiltrating cells are a hot topic in current research. There is growing evidence that high tumor‐associated macrophage infiltration is associated with disease progression and poor OS in patients with cancer. Multiple studies point to the need to identify molecular targets of immune‐infiltrating cells to develop therapies that target these harmful tumor‐infiltrating bone marrow cells.^34^ The correlation of the STAT family with various immune‐infiltrating cell types was explored using the TIMER database and the ssGSEA immune infiltration algorithm. The ssGSEA immune infiltration algorithm was used to establish that STAT1 was positively correlated with aDC, Th1 cells, and macrophages and negatively correlated with NK CD56bright cells, NK cells, and pDC. Similarly, STAT1 and STAT2 are correlated with various immune cell types. Meissl et al. found that STAT1 is essential for NK cell maturation and NK cell‐dependent tumor surveillance, and STAT1 loss‐of‐function and gain‐of‐function mutations lead to impaired NK cell cytotoxicity. This is in general agreement with our study, in which mutated STAT1 in endometrial cancer was negatively correlated with NK cells.^35^ Simultaneously, it has also been suggested that STAT1 signaling needs to be tightly controlled, with neither reduced nor excessive pathway activation being beneficial for NK cell maturation and function.^36^ It has been shown that IFN‐γ activates Jak/STAT1 signaling and promotes STAT1 phosphorylation, which leads to M1‐like macrophage polarization. RNA‐binding motif 4 acts as a cofactor for YTH N6‐methyladenosine RNA‐binding protein 2, which induces degradation of m6A‐modified STAT1 mRNA, thereby inhibiting glycolysis and M1 macrophage polarization.^37^ STAT6 was positively correlated with Th17 cells, CD56bright cells, and neutrophils and negatively correlated with Th2 cells, macrophages, and Th1 cells. Th2 differentiation is dependent on transcription factors, such as GATA3 and STAT6, which initiate the secretion of IL‐4, IL‐5, and IL‐13 by Th2 cells. STAT6 is a Th2‐inducible transcriptional activator that regulates epigenetic modifications and coordinates maturation of peripheral Th2 cells. The function of Th2 cells is impaired when STAT6 is mutated in tumors.^26^ STAT6 deficiency is associated with a higher cytotoxic NK cell activity. IL‐4/STAT6 activation and STAT6 deficiency in tumors result in increased cytotoxicity of NK cells.^36^ STAT1 and STAT6 showed opposite correlations with various immune cell types. This suggests that STAT1 and STAT2 have similar roles in UCEC prognosis, whereas STAT1 and STAT6 have opposite roles in UCEC prognosis. Activated STAT3 promotes the expression of proangiogenic and immunosuppressive factors in cancer cells and is essential for tumor progression. STAT3 is also activated in infiltrating immune cells, which enhances immunosuppression. Numerous cytokines signal by stimulating STAT3 or STAT5. This suggests that these two transcription factors play key roles in regulating T‐cell function. Activation of STAT3 and STAT5 may have beneficial or detrimental effects on the antitumor response, depending on the targeted T‐cell type.^38^ STAT3 activation in the tumor stroma is associated with impaired tumor immune surveillance of NK and CD8+ T cells.^36^ Ectopic expression of STAT5A enables the expansion of tumor‐specific CD4+ T cells and triggers antitumor CD8+ T‐cell responses.^39^ The results from the TIMER database showed that all STAT family members were positively correlated with neutrophils and dendritic cells. The level of immune cell infiltration differed according to immune infiltration software.

The present study had certain limitations. We illustrated the expression level and prognosis of the STAT family in endometrial cancer and its relationship with the UCEC immune infiltration level using multiple databases. However, this has not been validated through basic experiments. Bioinformatic analysis suggests that the STAT family has potential prognostic markers for UCEC as its therapeutic targets, particularly STAT1 and STAT6. However, definitive conclusions cannot be drawn. This study initially explored the possible molecular mechanisms and signaling pathways of STAT family‐related genes in UCEC. However, the specific functions of individual STAT family members and the regulation of immune‐infiltrating cells in endometrial cancer require further investigation.

## 5 CONCLUSIONS

The STAT family is associated with the prognosis and level of immune infiltration in endometrial cancer. High STAT1 expression and low STAT6 expression may be detrimental factors in UCEC prognosis. STAT‐related genes play a critical role in signal transduction and transcriptional activation and are involved in tumor development. The STAT family is expected to be a prognostic marker, and the level of immune infiltration, a therapeutic target, for endometrial cancer.

## CONFLICT OF INTERESTS

The authors declare that they have no conflict of interest related to this study.

## AUTHOR CONTRIBUTIONS

Zhou Xinying made significant contributions to this work. Dai Haiyan and Zhang Hu are corresponding authors of this article. All authors listed have made a substantial, direct, and intellectual contribution to the work, and approved it for publication.

## ETHICS APPROVAL

The data used in this study are publicly available and allow unrestricted reuse through open licenses. All datasets in this study are available for download from public databases, including TCGA, UALCAN, HPA, GEPIA, GeneMANIA, Kaplan–Meier Plotter, cBioPortal, String, David, and TIMER databases. The patients involved in the databases are ethically approved, and users can download the relevant data for research and publication free of charge, so ethical approval is not required.

## Data Availability

The data that support the findings of this study are openly available in TCGA database at https://portal.gdc.cancer.gov/.
